# Growth surveillance indices and Kashin-Beck Disease in children

**DOI:** 10.25122/jml-2021-0125

**Published:** 2022-02

**Authors:** 马玮娟 (Ma Wei Juan)

**Affiliations:** 1. Independent researcher, Baoji, Shaanxi, China

**Keywords:** Selenium, manganese, calcium, growth, Kashin-Beck disease, MS – value of Mn/Se, CS – value of Ca’/Se, MC – value of Mn/Ca’

## Abstract

Selenium, manganese, and calcium are necessary elements for maintaining normal growth and skeleton formation. Kashin-Beck disease mostly occurs in children, resulting in deformities, dwarfism, and disabilities. Selenium deficiency was considered a risk factor in China, while manganese was reportedly involved in it in Russia. Single-element regulation cannot be used in diagnosis because of unclear boundaries in patients compared to healthy individuals. In this study, new indices of elements were designed to predict the status of disease. MS (Mn/Se), CS (Ca’/Se), and MC (Mn/Ca’) values were designed, and prediction formulas were generated by comparing healthy children with those with Kashin-Beck disease via multiple linear regression analysis and discriminant analysis. In the disease group, 42.86% of patients had positive MS, CS, and MC values, and 57.14% of patients had positive MS and CS values. In the treatment group, the patients presented improved indices. In the prediction group, subjects with negative clinical criteria features were predicted by new indices, and 26.67% of them presented with positive MS, CS, and MC values, whereas 40.00% had positive MS and CS values. The 3D model of MS, CS, and MC refers to the setup of elements. The MS, CS, and MC indices are helpful in disease prediction, diagnosis, prognosis, and surveillance. The distribution model of the indices could serve in the growth surveillance of children.

## Introduction

Regulation and balance of elements occur in the body throughout human life. Breaking away from that balance can result in disease. Kashin-Beck Disease (KBD) mostly occurs in children and causes damage to epiphyseal growth cartilage and articular cartilage, mostly resulting to lifelong deformity and disability due to joint enlargement and stiffness, even after treatment. Abnormal mineralization and calcification resulted in enlarged deformities in the body. In contrast, chondrocytes stimulate apoptosis, necrosis, and proliferation. Therefore, an earlier onset indicates a more severe deformity.

Selenium (Se) deficiency was considered as risk factor of KBD in China and has been previously studied [1–8]. However, Se levels have never been considered as diagnostic criteria, because it was difficult to find the clear boundary between healthy and non-healthy patients. Manganese is reportedly involved in KBD cases in Russia [9]. However, to date, the clinical diagnostic criteria have been positive X-ray features, indicating a lag in progress, thereby affecting prevention and control. In this study, three new indices in prevention and control were reported with elements in the hair of children.

## Material and Methods

Hair samples from 1–2 cm of the skin side were shaved from the back of the head, and treated according to the standard procedure [10]. Elemental components in the hair of all subjects were analyzed by inductively coupled plasma-mass spectrometry [10] (ICP-MS; Sciex Elan 6000, Perkin-Elmer; hair samples were assayed at the Biological Trace Element Research Institute, University of California, USA). All data were analyzed using SPSS (version 18.0).

All analyzed subjects were obtained from a prevention evaluation study on KBD decades ago. In 1999, Se supplementation was reported to be effective in children with KBD [11]. A total of 39 elements were analyzed via a logistic regression study with 150 male children aged 6–14 years. Female children were not included because their physiological situations may influence some elements. Data from one supplemented group were missing, including 30 KBD cases treated with Se salt in 1/60000 proportion. In this study, the remaining 120 subjects were divided into four groups. Thirty healthy children with negative radiographic features were included in the healthy group (group 1) from Chang’an County in the non-KBD area. In the KBD area, 30 children with negative X-ray features were selected as the inner control group (group 2) from Bin County. Among 60 KBD children in Bin County with positive X-ray features, 30 who were not involved in any treatment comprised the KBD group (group3); the other 30 patients had been treated with 1 mg of Na_2_SeO_3_ as an Se supplement weekly for 6 months, and they were assigned to the Se supplement group (group 4). In the KBD group, two cases had negative records of elements, but only positive X-ray records were treated as missing data. Thus, 28 patients in the KBD group were included in this study. The clinical inclusion criterion was the national “Diagnostic Criteria for Kashin-Beck Disease” in China [12], including radiological changes mainly with irregular or sclerosis of the provisional calcification zone in the metaphysis, distal end phalanx alteration, epiphysis changes, and physical symptoms mainly with enlarged knuckle or joint, limited extension and bend, shortening, or deformity.

## Results

### Indices in the subjects

Se, manganese (Mn), and calcium (Ca) levels were separately analyzed in the KBD and healthy groups. In the KBD group, there was a significant correlation between Mn and Ca at 0.01 level, the Pearson correlation coefficient was 0.932. The other two correlations in the KBD group were positive without significance. In contrast, the correlation between Se and Ca in the healthy group was negative. Therefore, Mn and Ca are involved in the KBD process directly in symptom alteration because Mn is a necessary element in mineralization, and Ca contributes to calcification. Both are important for bone formation. However, Mn and Ca, including their association with Se, have not been well evaluated in KBD in China. Se is involved in regulating skeleton formation. Se supplementation decreased the incidence of KBD but could not control it, as expected. Se, Mn, and Ca levels from each group are described in Table 1; however, there were no clear boundaries among the groups, and it was difficult to estimate the disease status in all children. Therefore, new indices of MS, CS, and MC are designed. The values of MS (Mn/Se) and CS (Ca’/Se) may evaluate the status of bone formation during growth, and MC (Mn/Ca’) is helpful in evaluating the balance of storage because they are involved in mineralization and calcification. All tested Ca values were divided by 100 and labeled as Ca’ before calculating the reported values. Elements in hair represent storage and constructed elements in the body. In this study, MS and CS values were clearly divided into healthy and KBD groups in the full range. The MC value crossed all groups, but the maximal result from the healthy group was 0.86, lower than 1.00 of the other groups (Table 2). The 99% confidence ranges of all values are calculated as a reference in Table 2. MS, CS, and MC among groups had a significance difference of 0.000 via the Kruskal-Wallis test.

**Table 1.** Description of detected elements.

	**Range (μg/g)**	**n**
**Ca1**	214.00–656.00	30
**Ca2**	188.00–718.00	30
**Ca3**	358.00–1662.00	28
**Ca4**	450.00–1389.00	30
**Mn1**	0.41–3.05	30
**Mn2**	0.62–13.33	30
**Mn3**	1.50–32.06	28
**Mn4**	1.80–18.72	30
**Se1**	0.28–1.58	30
**Se2**	0.04–0.31	30
**Se3**	0.04–0.58	28
**Se4**	0.04–0.98	30

1 – healthy group; 2 – inner control group; 3 – KBD group; 4 – KBD with Se supplementation group; n – number of subjects.

**Table 2.** Description of indices.

	**Minimal**	**Maximal**	**99% confidence interval**	**Ԁ**	**S**	**n**
**MS1**	0.50	5.13	1.30–2.24	1.74	1.09	30
**MS2**	2.02	333.25	25.29-82.84	47.85	65.38	30
**MS3**	16.14	616.00	79.20–253.80	154.55	168.59	28
**MS4**	5.17	107.25	17.76–43.10	28.64	27.77	30
**CS1**	1.36	18.34	5.28–8.36	6.71	3.28	30
**CS2**	8.03	179.50	46.60–89.50	65.18	48.00	30
**CS3**	19.93	415.50	102.07–204.61	145.61	98.22	28
**CS4**	8.69	154.25	21.62–57.76	35.39	38.34	30
**MC1**	0.09	0.86	0.21–0.35	0.28	0.17	30
**MC2**	0.16	1.86	0.48–0.83	0.64	0.38	30
**MC3**	0.37	1.95	0.73–1.13	0.91	0.43	28
**MC4**	0.31	1.83	0.70–1.03	0.86	0.37	30

MS – value of Mn/Se; CS – value of Ca'/Se; MC – value of Mn/Ca'; 1 – healthy group; 2 – inner control group; 3 – KBD group; 4 – KBD with Se supplementation group; Ԁ – average; S – standard error; n – number of subjects.

### Prediction of disease by MS, CS and MC

Groups 3 and 4 were diagnosed as X-ray positive when included in the study. Alterations in all biochemical indices must occur earlier than the X-ray results. However, biochemical indices from blood and urine are influenced daily by the diet. Elements in hair could represent their storage status in the body in the past weeks or months and could serve as reliable indices. The minimal values of MS and CS from the KBD group were used as the standard to evaluate the inner control group, to find the possible patients or subjects at risk who have negative results on radiography, but positive in these indices (Table 3). MS values >16.14 and CS values >19.93 were considered positive. The maximal MC value from healthy individuals was used as the standard, and values >0.86 were considered positive. In addition, the evaluation of patients with Se supplementation indicated the Se regulation function and treatment status. Group 4 had fewer positive indices than Group 3. All X-ray-positive KBD patients were divided into two groups according to these values: 12 (42.86%) patients had three positive indices, and the other 16 (57.14%) patients had positive MS and CS values. In the inner control group, 8 (26.67%) subjects had three positive indices, 12 (40.00%) subjects had positive MS and CS values, 5 (16.67%) subjects had positive CS values, and 1 (3.33%) subject had positive MC (Table 3, a).

**Table 3.** Evaluation and prediction of patients by indices.

		**X-ray**	**MS+CS+MC**	**MS+CS**	**MS+MC**	**CS+MC**	**CS**	**MC**	**MS**
**Healthy**	0	0	0	0	0	0	0	0
**KBD**		28	12 (42.86%)	16 (57.14%)	0	0	0	0	0
**KBD+Se**	30	6 (20.00%)	6 (20.00%)	3 (10.00%)	0	2 (6.67%)	3 (10.00%)	0
**Inner control**	**a**	0	8 (26.67%)	12 (40.00%)	0	0	5 (16.67%)	1 (3.33%)	0
	**b**	0	4 (13.33%)	1 (3.33%)	0	0	3 (10.00%)	5 (16.67%)	0
	**c**	0	8 (26.67%)	11 (36.67%)	0	0	0	1 (3.33%)	0
	**d**	0	8 (26.67%)	11 (36.67%)	0	0	0	1 (3.33%)	0

MS – value of Mn/Se; CS – value of Ca'/Se; MC – value of Mn/Ca'; Ԁ_m_, average Mn level; Ԁ_s_ – average Se level; Ԁ_c_ – average Ca' level; a – inner control group with full range of KBD; b – inner control group with 99% confidence interval of the average KBD; c – prediction of inner control group by multiple linear regression; d – prediction of inner control group by classical and Bayes discriminant.

KBD causes deformity in patients, and the full range of analyzed indices from KBD is more priority than the 95% or 99% confidence range in diagnosis. Less subjects were evaluated as positive when comparing their results with a lower value of 99% confidence range of KBD (Table 3, b).

In multiple linear regression analysis, the healthy group and KBD group are datasets of equation (1), coefficients and constants are significant at the 0.01 level. Data from the crowd can be analyzed using equation (2). To predict the inner control group via equation (1), 20 positive and 10 negative subjects were generated (Table 3, c). In discriminant analysis, Wilks’ lambda was significant at 0.01 level, and the same result was obtained as in equation (1) (Table 3, d). Both regression and discriminant analysis are capable of reporting positive or negative results in the study but cannot recognize which index is positive. The results in lines c and d in Table 2 are counted by classifying positive subjects according to the previous standard in line a. Eight (26.67%) subjects had three positive indices, 11 (36.67%) subjects had positive MS and CS, and a subject (3.33%) had positive MC. Thus, 20 (Table 3, a) or 19 (Table 3, c, d) subjects from the inner control group who expressed with at least two positive indices are in risk exposure and are suspected to be potential X-ray positive patients by the next half-year. They should be included in preventive treatment or surveillance.



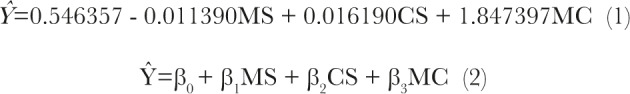



The MS, CS, and MC values are more sensitive than the single element test in directly comparing the study between groups. MS and CS enlarged the distance between healthy individuals and patients in the analysis. Therefore, in a large population survey, it is possible to calculate the risk ratio (rr), compare it between groups, and be predicted using equation (3). In this condition, the average content of each element in the crowd is close to that of the healthy one. In the current study, regression analysis generated the same prediction when the equations based on MSrr, CSrr, and MCrr. Data not written in this paper.



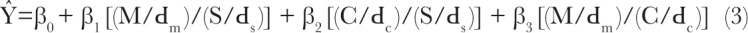



**Figure 1.**
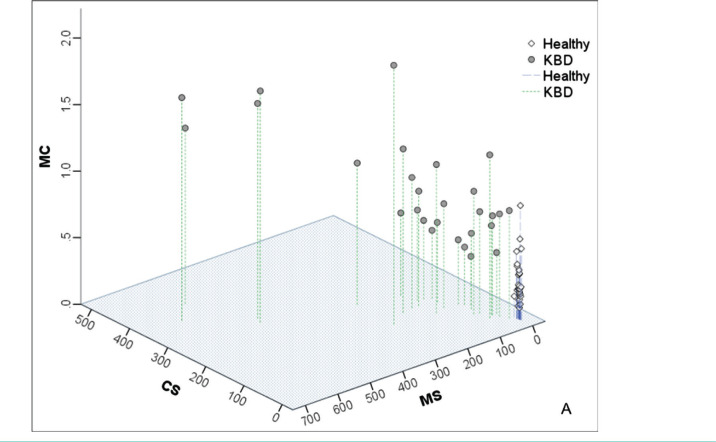

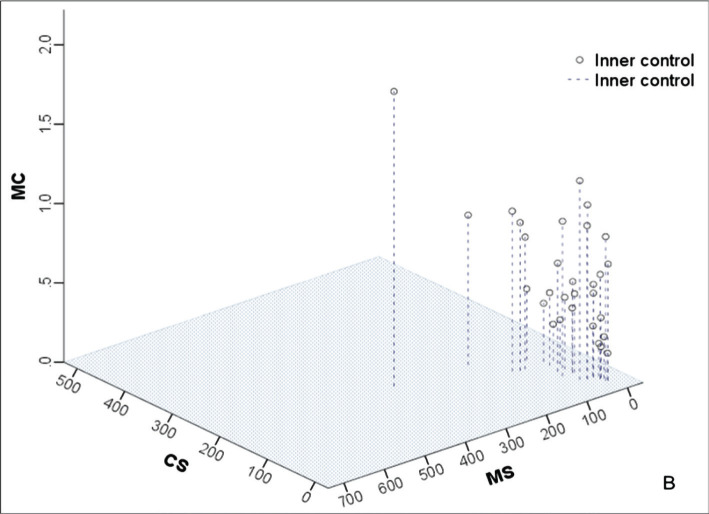
Distributions of subjects. A – 3D distribution of index values in the healthy and KBD groups; B – 3D distribution of index values in the inner control group; MS – value of Mn/Se; CS – value of Ca'/Se; MC – value of Mn/Ca'.

Setting CS, MC, and MS values as X, Y, and Z axes separately, three-dimensional distributions of data were generated using SPSS (Figure 1 AB). This is the basis for setting up the formula and prediction. MS, CS, and MC values in the healthy group were distributed in a limited range, gathering, and nearing to origin. In the KBD group, all indices expanded away from the healthy group due to lower Se, which was caused by increased MS and CS values, and some patients also had increased MC. In the inner control group, some subjects’ values were located in the healthy group’s range, while the others were similar to those in the KBD group. The distributions of the subjects in the inner control are consistent with the predictive results in Table 2.

### Distribution model of MS, CS and MC

**Figure 2.**
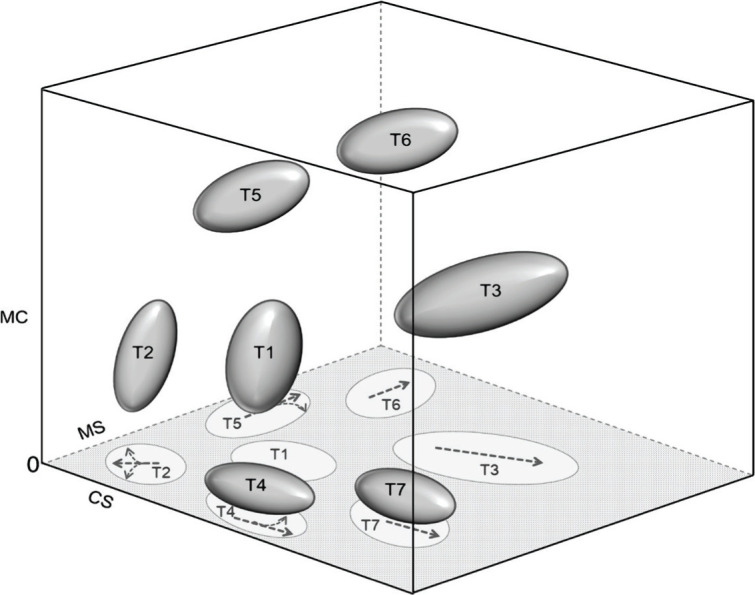
3D distribution model of indices’ values in 7 types (T1–T7). MS – value of Mn/Se; CS – value of Ca'/Se; MC – value of Mn/Ca'.

Three elements are involved in the indices. Selenium deficiency is an important risk factor that causes lower MS and CS in KBD, and some patients have higher MC. Patients could be clearly separated from the healthy group in the 3D distribution model of three indices, similar to type 3, separate from type 1 (Figure 2). In type 3, the data could expand to further locations with increased MS and CS. Theoretically, the influence of Mn and Ca can be predicted by the MS, CS, and MC values. Mn deficiency causes lower MS and MC; thus, data are distributed as type 4 compared to healthy individuals (type 1). Ca deficiency could cause lower CS and higher MC; thus, data are distributed as type 5 compared to type 1. Both Mn and Ca deficiencies cause lower MS and CS, as in type 2 distributions, and the data expand to both axes. Over absorption and storage Mn results in higher MS and MC and can be distributed as type 6. Over-absorption and storage of Ca results in lower MC and higher CS, and the data are distributed as type 7. There are two types not mentioned in the 3D model: one is over Se, which may overlap with type 2 in the data distribution. The other is that both Mn and Ca may overlap with type 3 in the data distribution. In these situations, three elements should be added directly as another three independent elements into the equation to separate them with type 2 or type 3 separately.

According to a previous study, the KBD area in Russia was over Mn [9, 13], but Ca and Se were not mentioned at the same time. If patients absorb an excess of both Mn and Ca, the distribution is similar to that of type 3, equation (1) is still effective. In addition, KBD patients caused by Se deficiency or over Mn and Ca could be separated by adding Se, Mn, and Ca directly as independent factors in the equation. Another method to separate excessive Se from type 2, or separate excessive Mn and Ca from type 3, is to analyze the data by steps: the first step is to separate alteration data from healthy by equations (2) and (3), and the second step is to set a new equation with three elements detected results as independent directly to divide subgroups in alteration data.

## Discussion

X-ray detection has been the current national diagnostic in clinics in China [12]. Similar to the inclusion in this study, all patients were diagnosed via X-ray detection by a clinic physician. It is a lag diagnostic method compared to the alteration of element regulation. Many KBD patients suffer from deformities after treatment, because visible abnormal calcification line, absorption invasion, or over-proliferation of sub-cartilage-like tissue may cause permanent cartilage damage and are mostly irreversible. The indices of MS, CS, and MC have been well evaluated in inner control in this study, and their application in surveillance can protect children from KBD before cartilage damage. These indices are easily accepted by children and their parents and are more reliable than blood or body fluids in surveillance. In addition, other type of distributions of indices may serve in growth surveillance in different situations that are influenced by elements.

China and Russia are the two primary KBD areas in the world. Almost no patients have been reported in Russia in recent years. Regardless of whether the disease was controlled well or dismissed naturally in Russia, Mn was possibly causal to the phenomenon; however, Se deficiency was not mentioned [9, 13]. In China, one report has mentioned that Mn increased and Se decreased in the hair of children in endemic areas, but Mn was not considered a risk factor because several control groups were designed, and only one control group had a significant difference with KBD [4]. Another study reported Se deficiency in KBD, while some elements including Mn increased in hair but were not significantly different [11]. One report demonstrated that Mn levels were significantly increased in the hair of KBD children compared to that of controls, with no mention of Se levels [5].

There is a supposition in this study, patients from Russia could overlap or beside the distribution of Chinese patients in model, it’s up to they presented as over Mn only or both of Mn and Ca. If patients from the two areas have different risk factor exposure, this indicates that the possible causal may still be hidden. The earlier the patient was found to be, the more likely it was to control the disease, and more likely to be causal in etiology.

In the mechanistic research, all specimens were obtained from adult donors. The target tissue cartilage can be obtained in a joint replacement operation in aged adults. Although patients had positive X-ray features, it was difficult to collect their onset information. Some patients may not be in the active period of KBD; they have sequelae caused by cartilage damage and subsequent deformity. In this matter, age or other influences on KBD in adults must be considered when demonstrating the pathogenesis in research results. Thus, current in vitro studies on KBD in adults still need more proof to explain element regulation as to the alteration in children’s hair.

## Conclusion

The MS, CS, and MC indices in children’s hair are noninvasive, acceptable, and reliable indices in the prediction, diagnosis, prognosis, and surveillance of KBD, and are useful in growth surveillance of children in other situations. Children with positive indices should be advised to accept preventive treatment.

## Acknowledgements

### Conflict of interest

The author declares no conflict of interest.

### Authorship

马玮娟 (Ma Wei Juan) contributed in indices design, data collection, analysis and writing.
